# The presence of primary circulating prostate cells is associated with upgrading and upstaging in patients eligible for active surveillance

**DOI:** 10.3332/ecancer.2017.711

**Published:** 2017-01-10

**Authors:** Nigel P Murray, Eduardo Reyes, Cynthia Fuentealba, Socrates Aedo, Omar Jacob

**Affiliations:** 1 Hospital Carabineros of Chile, Nunoa, 7770199 Santiago, Chile; 2 Faculty of Medicine, University Finis Terrae, Providencia, 7501015 Santiago, Chile; 3 Faculty of Medicine, University Diego Portales, Manuel Rodrıguez Sur 415, 8370179 Santiago, Chile; 4 Hospital DIPRECA, La Reina, Santiago, Chile

**Keywords:** prostate cancer, active surveillance, circulating prostate cells

## Abstract

Active surveillance (AS) is a considered treatment option for men with low or very low-risk prostate cancer. However, on repeat biopsy some 25% were upgraded and recommended for active treatment. We compare the presence or absence of primary circulating prostate cells (CPCs) with the clinical pathological findings after radical prostatectomy in men fulfilling the criteria for active surveillance and the risk of reclassification for active observation (AO).

**Methods and patients:**

A single centre observational study was done involving 102 men who fulfilled the Epstein criteria for AS and underwent radical prostatectomy as mono-therapy for prostate cancer. The patients were classified according to the presence or absence of CPCs detected immediately before the prostate biopsy. Mononuclear cells were obtained by differential gel centrifugation of 8 mL of venous blood and CPCs identified using immunocytochemistry with anti-PSA and anti-P504S. A positive CPC test was defined as at least 1 PSA (+), P504S (+) cell detected/blood sample. The surgical specimen was analysed for Gleason score and pathological stage.

**Results:**

A total of 25 out of 102 (24.5%) men were upgraded based on the pathological findings of the surgical specimen. Among which 45 (44%) men were positive for CPCs. They were younger, 63.9 versus 68.1 years (p = 0.0148), had a lower frequency of pT2 or lower disease (64.4% versus 91.2% p <0.001), higher median Gleason scores (6 versus 5 p < 0.001) in both the biopsy and surgical specimens, and a higher frequency of upgrading 44% versus 9% (p < 0.001)

**Conclusions:**

In men fulfilling the criteria for AS, the presence of primary CPCs suggests a high risk for disease upgrade and therefore these men may not be ideal for observational therapy. Further studies with a larger population are warranted.

## Introduction

Active surveillance (AS) is a recognised initial treatment option for men with early stage low-grade prostate cancer. The option to delay or avoid definitive therapy avoids or minimises patient morbidity without compromising long term outcomes in appropriately selected patients [1, 2]. According to the Prostate Cancer Intervention Versus Observation Trial (PIVOT) [3], men with low risk disease defined as a prostate specific antigen (PSA) ≤ 10ng/mL, a Gleason score ≤ 6, and T stage 1 or 2a had no difference in mortality, i.e., in terms of all-cause mortality and prostate cancer specific mortality. There was als no difference in rate of progression to bone metastasis when assigned to radical prostatectomy or AO. The criteria for AO according to Epstein [4] are a diagnosis of prostate cancer with two or less of the 12 prostate biopsy cores positive for cancer, no single biopsy core with > 50% infiltration, and a PSA density < 0.15ng/mL. Using these criteria to select patients with 'insignificant disease' has a positive predictive value of 95% and a negative predictive value of 66% [5]. These men are actively followed up with repeat annual biopsies; the timing of intervention after the initial diagnosis is based on variables such as PSA kinetics, Gleason grade progression, patient preference and/or clinical or radiologic evidence of disease progression [1, 6]. An increase in the Gleason score at repeat biopsy is predictive of the time to active treatment and correlates with patient outcome [7]. It has been reported that Gleason score progression occurs in approximately 20% of men, more than 50% of cases occurring within two years of the initial diagnosis [8]. However, a similar increase is seen in men subjected to immediate repeat biopsy when entering an AO programme [9]. This short time interval in comparison with the long natural history of prostate cancer suggests that sampling error rather than tumour progression is probably the primary source of tumour upgrading in these men. The use of other biomarkers, such as CPCs, could be useful in re-defining these patients who could be more adequately treated by AS.

Early in prostate cancer there are at least one sub-population of cancer cells that disseminate firstly to the neurovascular structures and then into the circulation [10]. The majority of these cells are eliminated by host defense mechanisms or destroyed by shear forces as they circulate in the blood and lymph systems [11]. These cells defined as primary CPCs are not found in small volume low grade cancers [12].

We present a prospective study in men who complied with the Epstein criteria for AO but choose to undergo radical prostatectomy. We compared the presence or absence of primary CPCs with the pathological findings of the surgical specimen to determine if men CPC positive were more likely to have 'upgraded cancer'.

## Patients and methods

A single centre observational study of a cohort of 426 men who underwent radical prostatectomy as the sole treatment for prostate cancer between 2005 and 2012. The study was approved by the local ethics committee and complied with the Declaration of Helsinki.

For each patient with an indication for radical prostatectomy, after giving informed written consent, the following were recorded; date of prostatectomy radical, age with the following clinic-pathological findings:

- total serum PSA (ng/mL) at the time of diagnosis using the Siemens AdviaCentaurXR® assay
- prostate volume at the time of biopsy: A transrectal ultrasonography of the prostate was performed using an endocavity convex probe with a 6.5 MHz transducer (Hitachi, model EVP-V33). Measures of the tri-axial distances of the prostate were taken in its larger diameter and the total volume was calculated by the following formula: volume = 0.52 x transverse diameter x anteroposterior diameter x longitudinal diameter.
- The pathological study of the prostate biopsy and surgical specimen were performed by dedicated genitourinary pathologists. Both the biopsy and surgical specimen were classified according to the Gleason system.

- Classification of the prostate biopsy: the number of biopsy cores from a total of 12 that were positive for cancer, and the percent infiltration of the cores were registered.
- Classification of the surgical specimen: the following findings were recorded;
  - presence or absence of extra-capsular extension (ECE),
  - presence or absence of positive surgical margins. It being defined as one with cancer cells in contact with the inked surface of the specimen.
  - infiltration of the seminal vesicles and lymph nodes

The same dedicated uro-pathologist informed both biopsy and surgical specimens.

### Definition of Epstein criteria for active observation

Men with a prostate biopsy positive for cancer fulfilled the Epstein criteria for AO when the Gleason score was ≤ 6, there were two or less of the 12 biopsy cores positive for cancer with an infiltration of ≤50% in any one core, and a PSA density of < 0.15ng/mL [4].

**Criteria for upgrading:** a cancer was determined to be upgraded when in the surgical specimen the Gleason score was ≥ 7 and the pathological stage was 2b or greater.

### Detection of primary circulating prostate cells

Before the prostate biopsy all men had a 8 mL venous blood sample was taken and collected in a tube containing EDTA (Beckinson-Vacutainer®). Samples were maintained at 4ºC and processed within 48 hours. CPC detection was independently evaluated with the evaluators being blinded to the clinical details.

#### Collection of CPCs

Mononuclear cells were obtained by differential centrifugation using Histopaque 1077 (Sigma-Aldrich), washed, and re-suspended in a 100 *μ*L aliquot of autologous plasma. Around 25 *μ*L aliquots were used to make slides (silanised, DAKO, USA), were dried in air for 24 hours, and fixed in a solution of 70% ethanol, 5% formaldehyde, and 25% phosphate buffered saline (PBS) pH 7.4 for five minutes, and finally washed three times in PBS pH 7.4.

#### Immunocytochemistry

CPCs were detected using a monoclonal antibody directed against PSA, clone 28A4 (Novocastro Laboratory, UK), and identified using an alkaline phosphatase-antialkaline phosphatase based system (LSAB2, DAKO, USA), with new fuchsin as the chromogen. Positive samples underwent a second process with anti-P504S clone 13H4 (DAKO, USA) and were identified with a peroxidase based system (LSAB2, DAKO, USA) with DAB (3,3diaminobenzidinetetrahydrochloride) as the chromogen. A primary CPC was defined according to the criteria of ISHAGE (International Society of Hemotherapy and Genetic Engineering) [13] and the expression of P504S defined according to the consensus of the American Association of Pathologists [14]; as a cell expressing both PSA and P504S and detected before definitive treatment for prostate cancer. A test was considered positive for primary CPCs when at least 1 cell/8 mL of blood was detected.

#### Statistical analysis

The analysis was performed using the programme Stata (Stata/SE 14.0 for Windows, Stata Corp Lp, 20159, describing according to the nature and distribution of the quantitative and ordinate variables with measurements of central tendency (mean and median) and of dispersion using the interquartile range (IQR) and standard deviation (SD). The Shapiro-Wilk test was used to define the null hypothesis with respect to the normal distribution. The nominal dichotomous variables were described as proportions with their respective confidence intervals.

Age, total serum PSA, percentage of positive biopsy cores, the Gleason Score of prostate biopsy, the Gleason Score of the surgical specimen, pathological stage T2a or less, extracapsular extension, surgical margins, seminal vesicle and lymph node infiltration according to its statistical were compared to the absence or presence of primary CPCs.

Student's t-test and Mann-Whitney test was used to compare quantitative variables. Hypothesis testing to compare two population proportion and Fishers´ Exact tests were used to compare frequencies on nominal variables. A P-value of ≤0.05 was taken to signify statistical significance and all tests were two tailed.

#### Ethical considerations

The study was approved by the local ethics committee and fully complied with the Declaration of Helsinki and its modifications.

## Results

Of the 426 men with an initial prostate biopsy positive for prostate cancer, 102/426 (23.9%) fulfilled the criteria for AO. These 102 patients formed the study population.

The 102 men showed a symmetrically age distribution with mean ± SD of 66 2± 8.6years. The total serum PSA (median: 5.16; IQR: 2.37), the number of positive biopsy cores (median:1; IQR: 1), median percentage infiltration 5% (IQR: 5%), the Gleason Score of biopsy (median 5; IQR: 1) and Surgical Specimen Gleason Score (median:5; IQR:2) showed asymmetrical distribution. The p-values of the Shapiro-Wilk test were 0.15 or more for age, Gleason Score of first biopsy and Surgical Specimen Gleason Score.

For the population total, 25 (24.51%; CI95%: 16.16–32.86)were disease upgraded based on the results of the surgical specimen. Among them 21 men (20.59%;CI 95%: 12.74–28.44) had pathological stage 2b or greater; extra-capsular extension was observed in 17men (16.67%;CI95%: 9.43–23.90); a positive surgical margin was present in one patient (0.98;CI 95%: 0–2.90). All men showed absence of both seminal vesicle invasion and lymph node infiltration.

Men positive for CPCs were older (p = 0.0148), had a higher median PSA (p = 0.0056), a higher median Gleason score in the prostate biopsy (p < 0.001) and surgical specimen (p < 0.001), fewer pathological stage 2a or less (p < 0.001) and higher frequency of extracapsular extension (p = 0.006) and disease upgrade (p < 0.001) (see Table 1).

Men CPC positive had a frequency of upgrade of 44.44% versus a 8.77% for men CPC negative, with a difference for test Hypothesis Testing to compare two samples that showed a p < 0,0001 and power (alpha 0.01, two tail) of 91.34%.

Therefore CPC positive men showed a relative risk of 5.07 (CI 95%: 2.06–12.44) with an absolute risk difference of 35.67% (CI 95%: 19.40–51.94%). of being upgraded (Table 1).

## Discussion

AO in the context of low risk prostate cancer is an option which can help to avoid treatment with surgery or radiation in some patients with indolent prostate cancer, while delaying active treatment in others. The use of AO is dependent on developing methods for selecting patients with a high possibility of indolent disease. The use of variations of the Epstein criteria has been suggested along with a validated nomogram to predict indolent cancer. It is defined as a pathologically organ confined cancer with a volume of less than 0.5 mL and no poorly differentiated elements. The nomogram has a discrimination defined as good with an area under of the curve of 0.64–0.79 [15, 16].

The observed association between the presence of CPCs and the pathological findings at surgery of higher Gleason score and higher pathological stage allows us to suggest that primary CPCs are another risk factor for the detection of more advanced prostate cancer. The absence of an association between the detection of primary CPCs and seminal vesicle and lymph node infiltration by cancer could be explained in that these patients have been classified as low risk for advanced cancer. The relative risk of upgrading is five times higher in men CPC positive.

This observational experience allows us to suggest that the presence of primary CPCs should be considered a risk factor for upgrading in patients considered to have low-risk prostate cancer.

We recognise that our study population is small, however, that approximately 24% fulfilled the criteria for AO and of these patients approximately 24.51% were upgraded is consistent with the reported data [17]. That this upgrading was determined within six months of the initial biopsy supports the concept of sampling error at the initial biopsy which is a well described phenomenon [18, 19].

We have previously reported that men with CPC negative prostate cancer had low grade, small volume tumours [12], which is supported by these new data. What we consider more important is that the presence of primary CPCs is associated with a high frequency of disease upgrade or upstaging in men who fulfill the criteria for AO. A 40% of men CPC positive were upstaged based on the pathological findings of the surgical specimen. Thus as a pre-treatment biomarker the presence of CPCs suggests that AO may not be the most adequate treatment option. It is important to note that primary CPCs have a limited prognostic value, independent of the method used to detect them [20, 21], but that men CPC negative have an excellent ten year biochemical failure free survival of > 90% [22] when treated with radical prostatectomy.

Serum PSA is the main biochemical marker used for the prognosis and progression of prostate cancer [23], however, 25% of those patients experiencing disease progression while on active observation do not have a rise in PSA levels [24]. Thus primary CPC detection may be a better biomarker for selecting patients for AO as currently only 10% of men eligible for AO actually elect this treatment option [25].

Other biomarkers have been used to assess the risk of upgrading; low serum testosterone has been associated with upgrading, upstaging, unfavourable disease and positive surgical margins [26]. Lower serum testosterone levels have been associated with high grade prostate cancer and a higher stage at presentation [27, 28]. Obesity and the metabolic syndrome have been associated with a low serum total testosterone and unfavourable outcome [29]. In the same context, men with a higher body mass index have been reported to have higher rates of upgrading and upstaging [30]. Of mention what is interesting is that the frequency of primary CPC detection has been reported to increase with increasing body mass index [31].

The expression of urotensin II receptor has also been reported to be associated with upgrading [32].

The detection of CPCs is highly method dependent and has previously been reviewed [33]. However, the method described does not require high state technology and can be implemented in the routine immunocytochemical laboratory of a general hospital. Limitations of this study include those inherent in its observational design. To reduce this interobsever variation, it is recommended that the same pathologist inform both the biopsy and surgical specimen. A larger study population is still needed to confirm these results.

## Conclusions

In men fulfilling the criteria for AO but are positive for primary CPC detection, there is a high risk of disease upgrade, thus these men may not be ideal candidates for AO. Further studies with a larger population are thus warranted.

## Conflict of interests

Dr Nigel Murray has received consultancy fees from Viatar CTC, Boston, USA.

## Funding

The study was funded by a Hospital de Carabineros de Chile Research Grant.

## Figures and Tables

**Table 1.** Clinical pathological finding of men CPC positive and negative.

Characteristic	CPC negative n = 57	CPC positive n = 45	P-value Two Tail
Age (years) mean ± SD	68.07 ± 8.19	63.91 ± 8.69	0.0148^a^
PSA (ng/mL) Median; IQR	4.79;1.32	5.51;2.64	0.0056^b^
Positive biopsy cores (%) Median; IQR	5;1	10;5	< 0.001^b^
Gleason Score of first biopsy Median; IQR	5;1	6;1	< 0.001^b^
Surgical Specimen Gleason Score Median; IQR	5;1	6;1	< 0.001^b^
Surgical Stage T2a or minor n (%)	52(91.22)	29(64.4)	< 0.001^c^
Extracapsular extension n (%)	4(7.02)	13(28.89)	0.006^d^
Upgrade n (%)	5(8.77)	20(44.44)	< 0.001^c^

IQR = interquartile range;

a Student`s t-test assuming equal variance;

b Mann-Whitney test,

c Hypothesis testing to compare two sample populations
